# Q Fever in Southern California: 11 Patient Cases from a Community-Based Organization

**DOI:** 10.4269/ajtmh.19-0557

**Published:** 2019-11

**Authors:** Andre K. Johnson, Falguni R. Patel, Jon G. Persichino

**Affiliations:** Department of Internal Medicine; Riverside Community Hospital; Riverside, California; Department of Pharmacy; Riverside Community Hospital; Riverside, California; Division of Infectious Diseases; Riverside Medical Clinic; Riverside, California; E-mail: jon.persichino@rmcps.com

Dear Sir,

We read with great interest the case series of 20 patients with Q fever from a VA Medical Center in southern California by Akamine et al.^1^ We applaud the authors’ efforts to create greater awareness for an infection that is considered underreported in this region.

We identified 11 cases with Q fever at our community-based organization in southern California by a retrospective chart review from 2014 to 2019 (Table 1). On review, all of our patients presented with a febrile illness, and a vast majority of cases involved males (nine) with animal exposure, particularly to goats and cattle (seven). The geographic distribution of the cases favored urban areas (10) in Riverside and San Bernardino counties. In addition, six patients with acute Q fever presented with transaminitis (four), leukocytosis (three), and pneumonia (two). Complications of infection caused seven of our patients to be hospitalized. All patients were treated with oral doxycycline for 14 days, except for patient no. 2 who received a 1-year course of doxycycline and hydroxychloroquine sulfate for endocarditis. All of our cases received infectious diseases consultation and follow-up with subsequent complete recovery from infection.

**Table 1:** Summary of demographic and clinical features of Q fever cases

Patient no.	Age (years) and gender at diagnosis	Clinical presentation	Animal exposure	Date of symptoms	Date of first titer	Case classification
1	63, M	Febrile illness	Yes—goats	July 18, 2014	August 28, 2014	Acute_c_
2	59, M	Febrile illness with thrombocytopenia	Yes—goats	March 27, 2016	May 4, 2016	Chronic_c_
3	67, M	Febrile illness	Yes—cattle	March 25, 2018	March 28, 2018	Acute_p_
4	73, M	Febrile illness with transaminitis	Yes—goats	July 6, 2018	August 8, 2018	Acute_c_
5	43, M	Febrile illness with transaminitis and leukocytosis	No	August 18, 2018	August 30, 2018	Acute_c_
6	47, M	Febrile illness	Yes—goats/cattle	August 18, 2018	September 4, 2018	Acute_p_
7	30, M	Febrile illness	Yes—cats/dogs	August 22, 2018	September 17, 2018	Acute_c_
8	70, F	Febrile illness	No	November 19, 2018	January 7, 2019	Acute_c_
9	34, M	Febrile illness with pneumonia, transaminitis, and leukocytosis	No	January 22, 2019	January 27, 2019	Acute_c_
10	34, M	Febrile illness with transaminitis and leukocytosis	Yes—goats/cattle	February 22, 2019	March 11, 2019	Acute_c_
11	43, F	Febrile illness with pneumonia	Yes—goats/cattle	April 4, 2019	June 14, 2019	Acute_p_

Acute_c_ = acute infection, confirmed; Acute_p_ = acute infection, probable; F = female; M = male.

Of significant interest, our findings revealed that a greater proportion of cases (nine) occurred between 2018 and 2019, which is of unclear etiology. Symptom onset was most frequent during the spring (three) and summer (five), with a peak in August (three). In addition, exposure to goats and cattle was the predominant risk factor for infection in our patients than exposure to rodents, dogs, cats, and sheep as reported in other studies in southern California.^1,2^ We identified two female patients with Q fever as compared with none by Akamine et al.^1^ Another distinctive finding from other studies is almost half of our patients were diabetic (five) and of Hispanic/Latino (five) descent.^1,2^

Our cases highlight that Q fever may be a bigger threat than previously thought in Riverside and San Bernardino counties of southern California. Our findings may also provide early evidence for an increasing trend of infection in this region. Health-care professionals should be vigilant in screening for infection in patients particularly presenting with a febrile illness associated with transaminitis, leukocytosis, pneumonia, and exposure to goats and cattle. We agree with the authors’ recommendation for early infectious diseases consultation to expedite diagnosis and treatment that may improve outcomes and potentially prevent complications of the infection. Further studies with larger cohorts involving multiple health-care institutions in southern California are warranted to confirm our observations for greater public health awareness.
